# hsa_circ_0000520 Serves as a Prognostic Biomarker for Colorectal Cancer and Promotes in the Disease Progression

**DOI:** 10.5152/tjg.2024.24153

**Published:** 2024-12-01

**Authors:** Bingzhe Shi, Xiufen Lu, Wanli Ma, Chao Huang, Junyue Huo

**Affiliations:** Department of Anorectal Surgery, Xingtai People’s Hospital, Xingtai, China

**Keywords:** Colorectal cancer, hsa_circ_0000520, prognosis, miR-542-3p

## Abstract

**Background/Aims:**

Colorectal cancer (CRC) constitutes one of the prevalent malignancies within the gastrointestinal tract and serves as a primary contributor to cancer-related mortalities. This investigation sought to investigate the expression and prognostic significance of hsa_circ_0000520 in CRC and to evaluate its impact on the onset of CRC.

**Materials and Methods:**

The levels of hsa_circ_0000520 were measured via quantitative real-time polymerase chain reaction (qRT-PCR). To delve into the mechanism through which circ_0000520 impacts CRC and to assess the cellular behavior of CRC cells, a series of experiments including the CCK-8, transwell assay, flow cytometer assay, cell cloning formation, dual-luciferase reporter assay, and bioinformatics method were performed.

**Results:**

The expression levels of hsa_circ_0000520 were markedly elevated in CRC cells and tissue specimens, and this elevation was correlated with a low survival rate. hsa_circ_0000520 affected CRC cell function via the miR-542-3p/MYH9 axis, thus exacerbating cancer progression.

**Conclusion:**

hsa_circ_0000520 functions as a predictive biomarker for the prognosis of CRC and participates in its progression. hsa_circ_0000520 emerges as a new treatment strategy for CRC patients.

**Main Points**

- circ_0000520 is highly expressed in CRC.
- Elevated circ_0000520 is associated with a poor survival rate.
- circ_0000520 enhances the proliferation, migration, and invasion of CRC cells.
- circ_0000520 negatively regulates miR-542-3p.

## Introduction

Colorectal cancer (CRC) constitutes one of the prevalent malignancies within the gastrointestinal tract and serves as a primary contributor to cancer-related mortalities.^1^ Patients may exhibit a spectrum of manifestations, including subtle or overt rectal hemorrhage, alterations in bowel routines, anemia, or abdominal discomfort. Nonetheless, CRC is typically characterized by a lack of symptoms until it progresses to an advanced phase.^4^ Despite advancements in the treatment of CRC, regretfully, the outcomes for CRC patients remain less than optimal.^5^ Accordingly, it is essential to comprehend the fundamental mechanisms driving the progression of CRC, with the goal of enhancing diagnostic efficacy, therapeutic outcomes, and patient survival rates.

Circular RNAs (circRNAs) represent a novel category of bifunctional RNAs that serve both non-coding and possess limited protein-coding roles.^6^ Emerging research indicates that alterations in circRNA expression are involved in the onset of multiple cancer types, including gastric cancer,^9^ thyroid cancer,^10^ hepatocellular carcinoma,^11^ and cervical cancer.^12^ Recent investigations have revealed altered expressions of circRNAs in the blood/serum, cells, tumor tissues, and exosomes of CRC patients.^13^ Accordingly, circRNAs are prospective biomarkers for the diagnosis of CRC.

hsa_circ_0000520 has been reported to be abnormally expressed in various tumors, such as the level of hsa_circ_0000520 was substantially elevated in glioma tissues and corresponding cells.^14^ Sepideh Kadkhoda investigated the expression patterns of circRNAs that exhibited altered expression in CRC, revealing that hsa_circ_0000520 was overexpressed in this malignancy.^16^ In addition, Xu Lei observed that colorectal tumors exhibited significant expression of hsa_circ_0000520.^17^ To date, the prognostic role of hsa_circ_0000520 in CRC, as well as the underlying mechanisms that influence CRC progression, remains unelucidated.

This study investigated the correlation between hsa_circ_0000520 level and the prognosis of CRC and explored the mechanism of hsa_circ_0000520 involvement in CRC.

## Materials and Methods

### Research Object

A collection of 119 paired samples, consisting of CRC specimens and their corresponding adjacent non-tumorous tissues, was retrieved from patients who had been treated with surgical intervention at Xingtai People’s Hospital. The patients were asked to return to the hospital regularly for reexamination, and the patients were followed up for 60 months. All participants signed informed consent forms, and the collection of specimens was meticulously examined and granted approval by the Institutional Review Board of Xingtai People’s Hospital (approval no: 2016-0035, date: 22/6/2016).

### Cell Line and Cell Culture

Cell lines of human CRC (HT-29, SW480, SW620, and HCT116) were sourced from the Cell Bank of the Chinese Academy of Sciences (Shanghai, China). Normal human intestinal epithelial cells (HIEC-6) were sourced from the American Type Culture Collection (ATCC, Manassas, United States). HT-29 and HCT116 cells were cultured in McCoy’s 5A (Gibco, Carlsbad, Calif, USA). HIEC-6, SW480, and SW620 cells were cultured in DMEM (Gibco). The cells were maintained in an atmosphere of 5% carbon dioxide, with conditions set at a temperature of 37°C.

### Cell Transfection

Colorectal cancer cells were seeded into 24-well culture dishes. siRNA targeting hsa_circ_0000520 (si-circ_0000520) or a non-targeting control (si-NC) and a miR-542-3p inhibitor were introduced into the CRC cells using Lipofectamine 2000 (Invitrogen, Carlsbad, Calif, USA). 

### qRT-PCR

The TRIzol reagent was used to extract total RNA (Life Technologies, Carlsbad, CA). The integrity and concentration of the extracted specimens were assessed using a Nanodrop 2000 device (Thermo Fisher Scientific, Waltham, MA, USA). The RNA samples were initially converted into cDNA using HiScript II Q RT SuperMix (Vazyme, Nanjing, China). Subsequently, the cDNA was amplified and detected using ChamQ SYBR qPCR Master Mix (Vazyme, Nanjing, China). The expression levels were quantified utilizing the 2^−ΔΔct^ approach. The sequences of the primers are as follows:

hsa_circ_0000520 forward: 5’-GTCTGAGACTAGGGCCAGAGGC-3’.
hsa_circ_0000520 reverse: 5’-GACATGGGAGTGGAGTGACAGG-3’.
miR-542-3p forward: 5’-GCCGAGUGUGACAGAUUGAUA-3’.
miR-542-3p reverse: 5’-CTCAACTGGTGTCGTGGA-3’.
GAPDH forward: 5’-TCACCAGGGCTGCTTTTAAC-3’.
GAPDH reverse: 5’-TGACGGTGCCATGGAATTTG-3’.

### Cell Proliferation Assay

Colorectal cancer cell suspensions were inoculated into each well of a 96-well plate at a density of 2000 cells per well. Subsequently, at intervals of 0, 24, 48, and 72 hours post-seeding, a volume of 10 μL of the Cell Counting Kit-8 (CCK-8; Invitrogen) was incorporated into each well. The plates were then incubated for 2-4 hours at a temperature of 37°C. The absorbance values of the samples were measured using a microplate reader (Infinite M200, TECAN, Switzerland) at a wavelength of 450 nm. 

### Transwell Assays

Migration and invasion were executed using transwell plates or Matrigel-coated transwell plates. In line with the specified procedure, single-cell suspensions were inoculated into the upper sections of the transwell and incubated for a duration of 24 hours. Subsequently, the cultures underwent a washing process with PBS, followed by fixation with paraformaldehyde and subsequent staining with crystal violet. Thereafter, the migration and invasion rates were computed by performing cell enumeration across a minimum of 5 randomly selected fields, guided by the outcomes of crystal violet staining.

### Flow Cytometry Assay

Apoptosis in cells was quantified utilizing an Annexin V-FITC Kit (BD Biosciences, San Diego, NJ, USA). A suspension of 5 × 10^5^ cells was prepared in binding buffer (200 μL), followed by the addition of FITC Annexin V (5 μL) and propidium iodide solution (1 μL). Cell apoptosis was determined employing a flow cytometer.

### Clone Formation Assay

Following the process of cellular transfection, the cells were suspended and seeded into 6-well culture plates at a density of 1000 cells per well, and they were allowed to grow for a duration of 14 days. Subsequently, the colonies were subjected to PBS rinsing, followed by a 10-minute fixation with 4% paraformaldehyde and a 15-minute staining process using 0.1% crystal violet at ambient temperature. Subsequently, the colonies underwent an additional PBS wash, and their cell colony numbers were tallied by manual counting.

### Dual-Luciferase Reporter Assay

The wild-type circ_0000520 (WT-circ_0000520), mutant circ_0000520 (MUT-circ_0000520), wild-type MYH9 (WT-MYH9), and mutant MYH9 (MUT-MYH9) were created and inserted into the pmirGLO luciferase reporter vector (Promega, Madison, Wis, USA). These plasmids were subsequently introduced into the SW480 and SW620 cell lines concurrently with miR-542-3p mimics and mimics-NC. The relative luciferase activities were determined by employing the dual luciferase assay (Promega). 

### Bioinformatics Analysis

The datasets employed in this research are accessible to the public without any cost. At present, GEO is the most extensive and comprehensive gene expression repository founded by the National Center for Biotechnology Information (NCBI), which housed data regarding the expression of mRNA across various samples. To obtain microarray data specifically associated with CRC, a search was conducted using the keyword “RNA&CRC” within the GEO database. The adjusted *P*-value was represented as adj. P. Val, and genes were deemed differentially expressed if they satisfied the condition of having a log2 fold change (|log2FC|) greater than 0.5 along with an adj. P. Val of less than 0.05.

### GO (Gene Ontology) and KEGG (Kyoto Encyclopedia of Genes and Genomes) Enrichment Analyses

The target genes of miR-542-3p were identified by referring to the TargetScan database. A bioinformatics platform was utilized to integrate data from the GEO and TargetScan databases, generating a Venn diagram that depicted the overlapping mRNAs. The overlapping genes were subjected to GO categorization and KEGG pathway enrichment analysis. The GO framework classifies genes into 3 separate classes: molecular functions (MF), biological processes (BP), and cellular components (CC). Additionally, KEGG pathway analysis was performed to examine the biological pathways in which these genes are involved.

### Statistical Analyses

Values are presented as the mean ± SD and analyzed via an independent *t-*test, 1-way analysis of variance (ANOVA), or 2-way ANOVA. Statistical analyses were conducted using GraphPad Prism version 9.0 (GraphPad Software, La Jolla, CA) and SPSS version 26.0 (IBM SPSS Corp.; Armonk, NY, USA). A Kaplan-Meier survival analysis was performed to examine the correlation between hsa_circ_0000520 and the overall survival of patients. Cox regression analysis was employed to determine prognostic factors influencing the prognosis of patients. A *P*-value of less than .05 was deemed statistically significant.

## Results

### Variation in Expression of hsa_circ_0000520 in Colorectal Cancer

**Figure 1.**
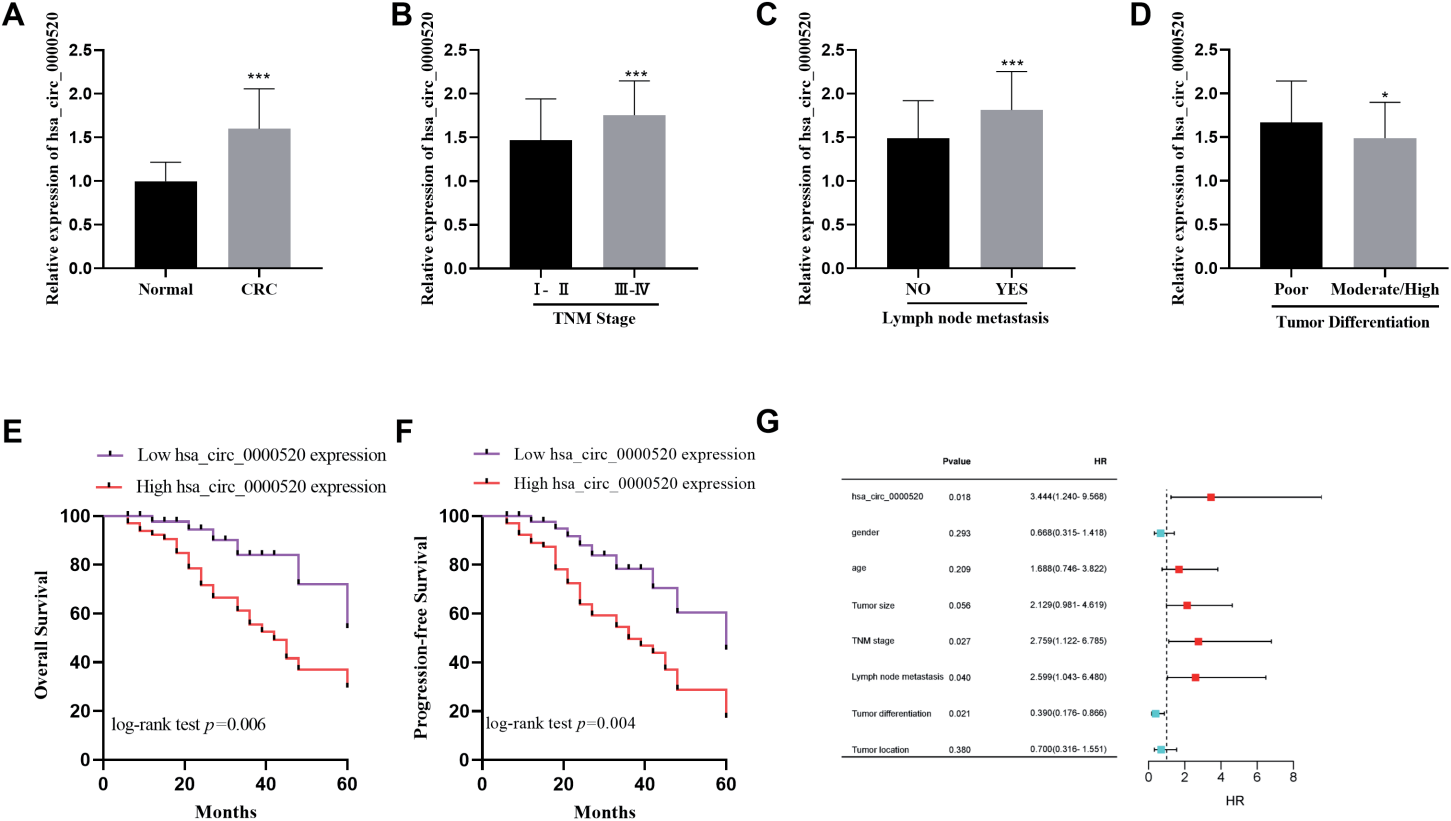
The level of hsa_circ_0000520 in patients with CRC and its prognostic value for CRC. (A) The level of hsa_circ_0000520 in CRC tissues was detected by qRT-PCR. (B) The expression level of hsa_circ_0000520 in stages I-II and III-IV was detected by qRT-PCR. (C) The expression level of hsa_circ_0000520 in patients with and without lymph node metastasis was detected by qRT-PCR. (D) The expression level of hsa_circ_0000520 in patients with different degrees of tumor differentiation was detected by qRT-PCR. (E) Kaplan-Meier analysis was employed to examine the correlation between hsa_circ_0000520 expression and overall survival of patients with CRC. (F) Kaplan-Meier analysis was employed to examine the correlation between hsa_circ_0000520 expression and disease-free survival of patients with CRC. (G) Forest map of multivariate regression analysis of CRC prognosis. ^*^P < .05, ^***^P < .001.

The findings demonstrated that hsa_circ_0000520 was markedly elevated in the tumor tissues (Figure 1A). Additionally, the expression of hsa_circ_0000520 was upregulated in patients with TNM stages III-IV compared to TNM stages I-II (Figure 1B). Similarly, the levels of hsa_circ_0000520 were observed to be elevated in patients presenting with lymph node metastasis, contrasting with those devoid of lymph node metastasis (Figure 1C). Moreover, a marked increase in hsa_circ_0000520 expression was detected in patients with poorly differentiated tumors, in comparison to those with moderately or well-differentiated tumor grades (Figure 1D). As indicated in Table 1, a substantial correlation was evident between hsa_circ_0000520 and the presence of TNM stage, the occurrence of lymph node metastasis, and the grade of tumor differentiation. Conversely, no significant correlation was identified between hsa_circ_0000520 and the patients’ gender, age, tumor size, or the tumor location.

### Effect of hsa_circ_0000520 on Prognosis of Colorectal Cancer Patients

The Kaplan–Meier survival analysis was conducted. The study indicated that patients demonstrating increased expression of hsa_circ_0000520 encountered a more adverse prognosis relative to those exhibiting decreased hsa_circ_0000520 expression levels (refer to Figure 1E). Additionally, the disease-free survival (DFS) rate was notably decreased in the group with increased expression of hsa_circ_0000520, as depicted in Figure 1F. The Cox regression analysis indicated that various factors, including TNM stage, lymph node metastasis, tumor differentiation, and hsa_circ_0000520 expression level, contributed to an elevated risk of mortality (refer to Figure 1G). The findings implied that higher expression of hsa_circ_0000520 potentially served as a predictor of CRC patient prognosis.

### Effect of Downregulated hsa_circ_0000520 on the Function of Colorectal Cancer Cells

**Figure 2.**
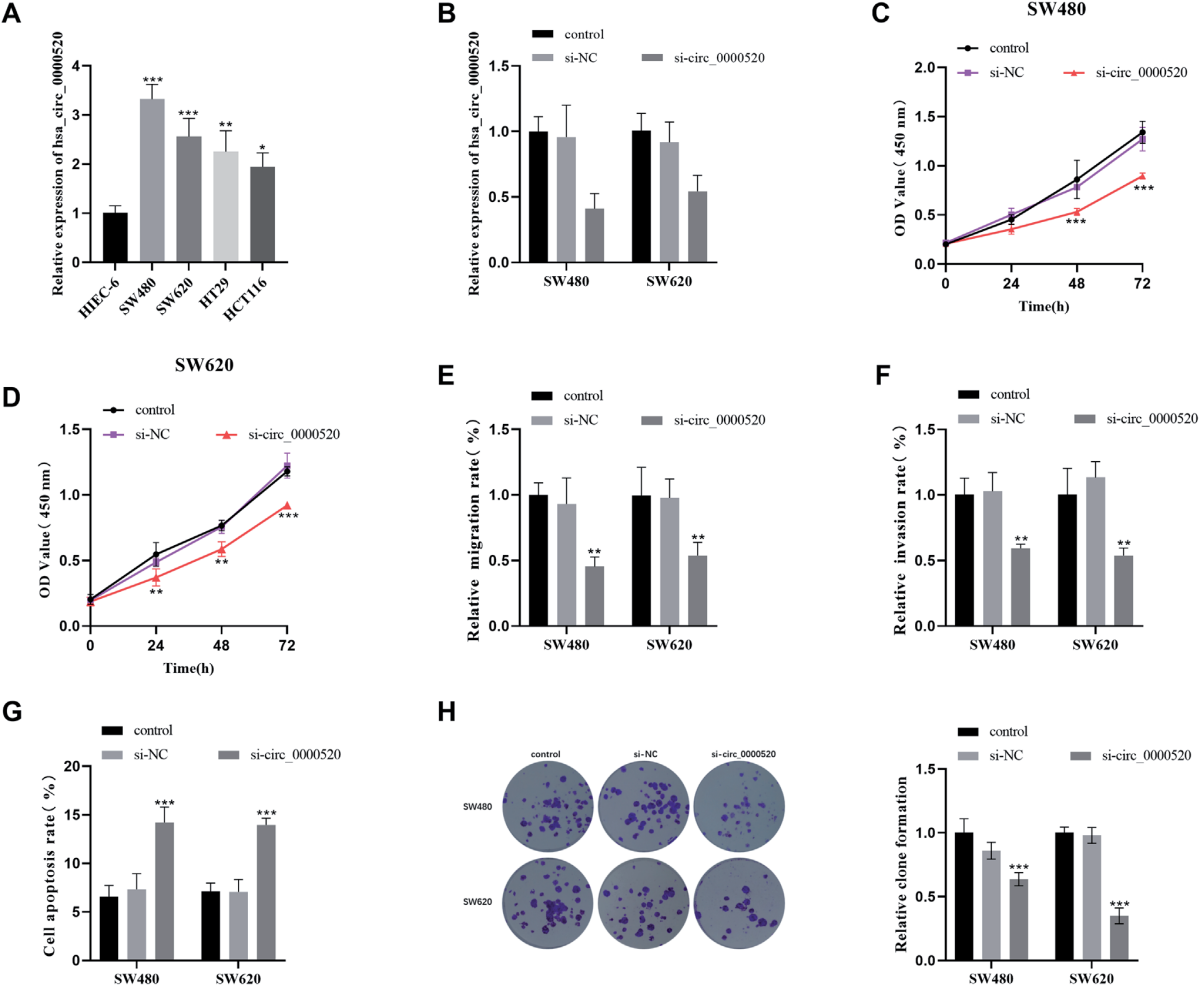
Effect of downregulating hsa_circ_0000520 on the function of CRC cells. (A) The expression of hsa_circ_0000520 in CRC cell lines was quantitatively detected by qRT-PCR. (B) The transfection efficiency of si-circ_0000520 was quantified via qRT-PCR. (C-D) CCK-8 assay was employed to assess cell proliferation after hsa_circ_0000520 knockdown. (E-F) The transwell assay was employed to assess the capacity of cell migration and invasion following hsa_circ_0000520 knockdown. (G-H) The flow cytometry assay and cloning experiment were employed to assess cell apoptosis and clone formation following hsa_circ_0000520 knockdown. ^*^P < .05, ^**^P < .01, ^***^P < .001.

The levels of hsa_circ_0000520 expression were noticeably increased within the CRC cells when contrasted with the normal cellular controls, with SW480 and SW620 showing particularly high expression levels (Figure 2A). Based on these findings, the SW480 and SW620 cell lines were selected for more in-depth examination within this study. By introducing si-circ_0000520 into the CRC cell lines, we detected a reduction in the levels of hsa_circ_0000520 expression within the cellular samples, as depicted in Figure 2B. The proliferation of si-circ_0000520-treated cells exhibited a significant decrease compared to the control group (Figure 2C and  D). The suppression of hsa_circ_0000520 corresponded to a reduction in the migratory and invasive capabilities of CRC cells (Figure 2E and  F). Furthermore, after si-circ_0000520 treatment, the apoptosis rate increased significantly (Figure 2G). Clone formation experiments showed that after circ_0000520 inhibition, the number of clones of both CRC cells was reduced compared to controls (Figure 2H).

### hsa_circ_0000520 Acts as a Sponge for miR-542-3p

**Figure 3.**
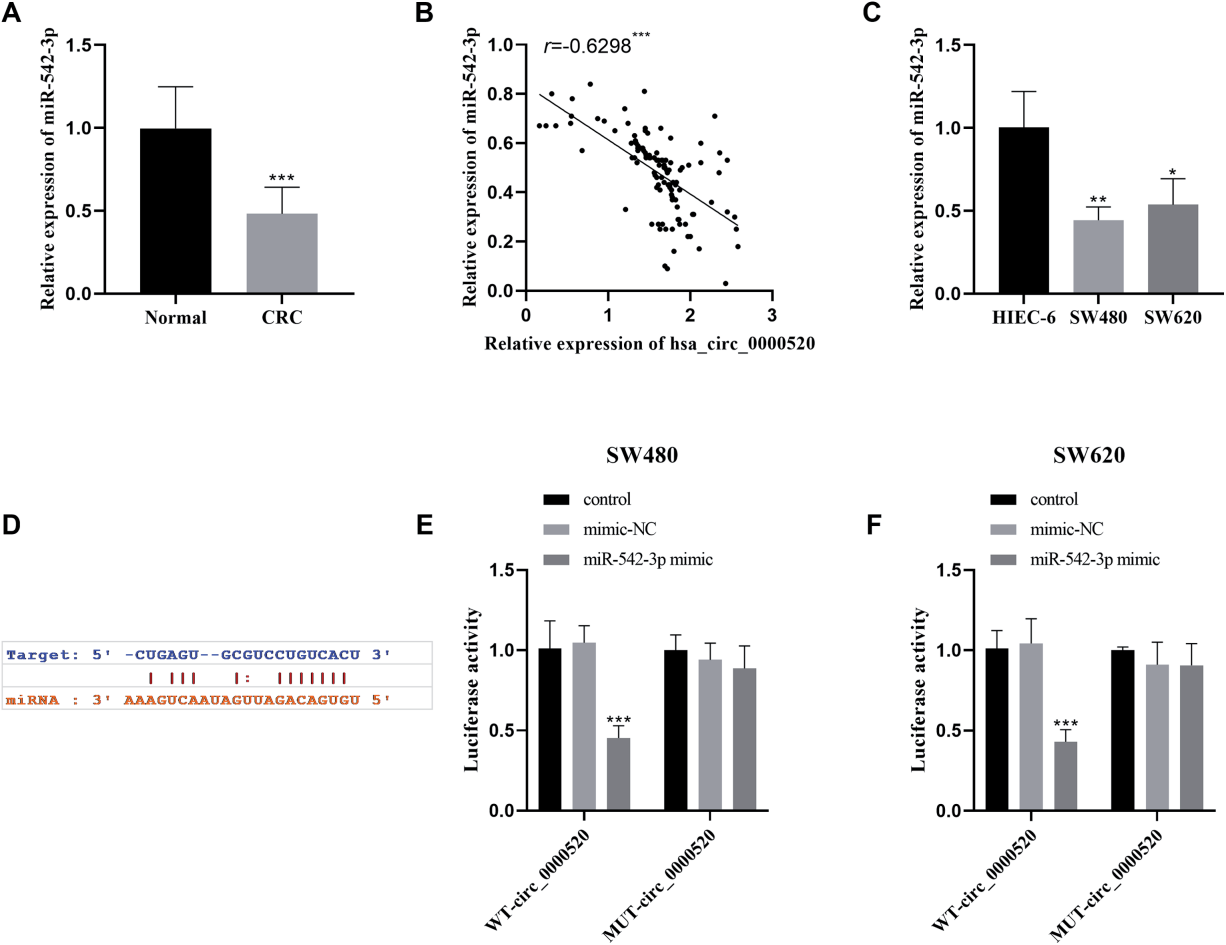
miR-542-3p is a target of hsa_circ_0000520. (A) The level of miR-542-3p in CRC tissues was detected by qRT-PCR. (B) The expression of miR-542-3p and hsa_circ_0000520 in tumor tissues was negatively correlated. (C) The expression of miR-542-3p in CRC cell lines was detected by qRT-PCR. (D) The sequence of the binding site between hsa_circ_0000520 and miR-542-3p was forecasted utilizing starBase. (E-F) The specific interaction between hsa_circ_0000520 and miR-542-3p was investigated using a dual luciferase reporter gene assay. ^**^P < .01. ^**^P < .01,^ ***^P < .001.

The results demonstrated a decrease in the expression level of miR-542-3p within the tumor specimens (Figure 3A), and a negative correlation was identified between the levels of miR-542-3p and hsa_circ_0000520 expression in the same tissues (Figure 3B). The expression levels of miR-542-3p within the CRC cell lines were significantly reduced when contrasted with normal colon epithelial cells (Figure 3C). The potential binding site between hsa_circ_0000520 and miR-542-3p was predicted by the starBase database (Figure 3D). In addition, the miR-542-3p mimic significantly decreased the luciferase activity of WT-hsa_circ_0000520 (Figure 3E and  F).

### miR-542-3p Inhibitors Partially Reverse the Effect Induced by hsa_circ_0000520 Knockdown

**Figure 4.**
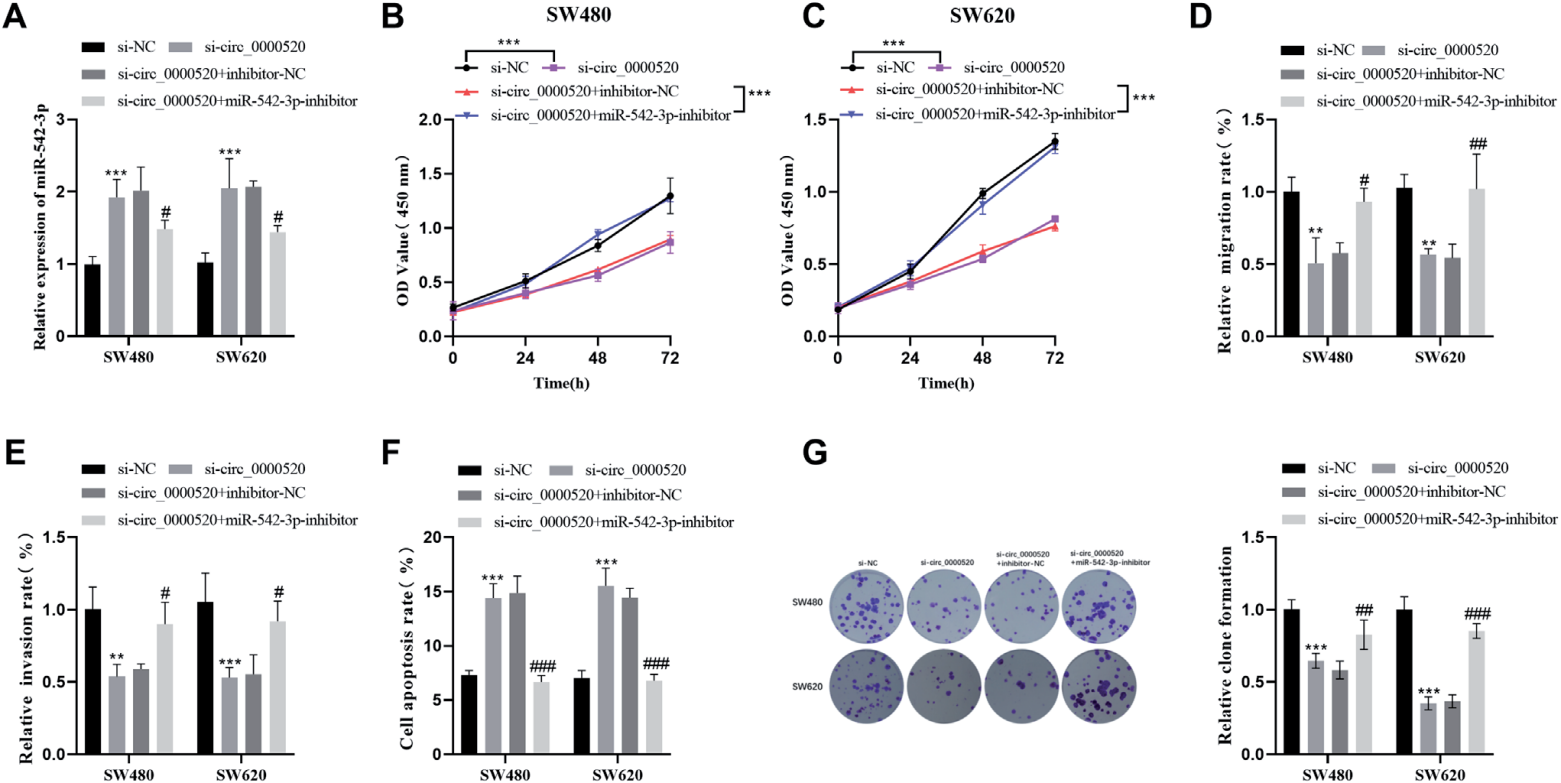
miR-542-3p inhibitor partially counteracts the effects of si-circ_0000520 on cellular function. (A) miR-542-3p level was quantified via qRT-PCR. (B-C) Cell proliferation was assessed using the CCK-8 assay. (D-E) The migration and invasion capability of cells was assessed by the transwell assay. (F-G) The flow cytometry assay and cloning experiment were employed to assess cell apoptosis and clone formation. ^**^P < .01, ^***^P < .001 vs. si-NC group;^ #^*P* < .05, *^##^P* < .01vs. si-circ_0000520+inhibitor −negative control (NC)group.

Knockdown of circ-0000520 led to an upregulation of miR-542-3p expression. This increase was partially reversed by the addition of a miR-542-3p inhibitor (Figure 4A). Additionally, miR-542-3p effectively countered the inhibitory effect of si-circ_0000520 on cell viability (Figure 4B and  C). The inhibition of cellular invasion and migration that was noticed after the decrease in hsa_circ_0000520 expression was lessened when miR-542-3p inhibitors were applied (Figure 4D and  E). At the same time, miR-542-3p was able to reverse the elevated rate of apoptosis and reduced number of clone formation induced by si-circ_0000520 (Figure 4F and  G).

### GO and KEGG Enrichment Analyses

**Figure 5.**
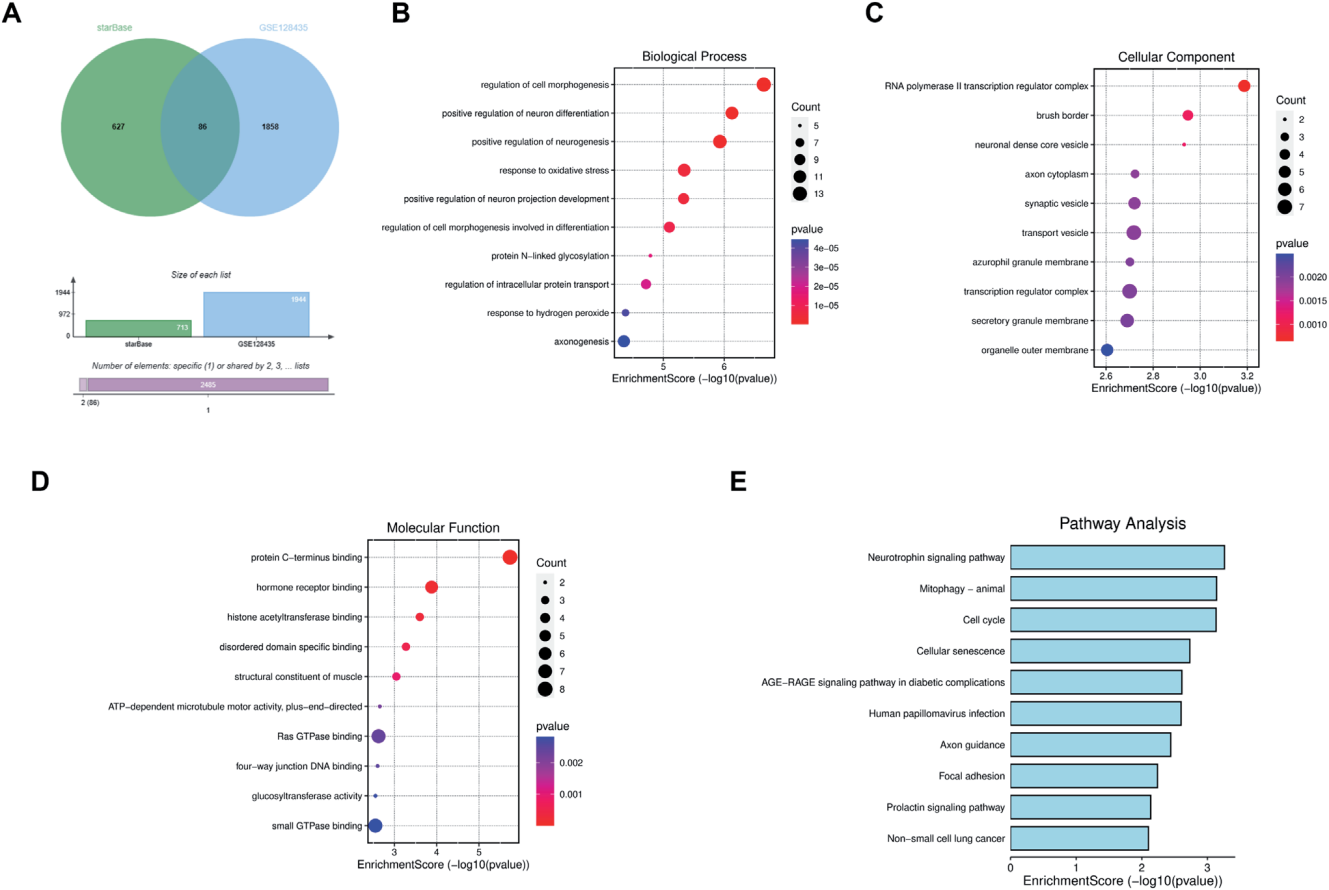
GO and KEGG enrichment analyses of CRC-associated mRNAs. (A) The target genes were identified using the starBase database and GEO database. (B-D) The overlapping genes were analyzed by GO analysis. (E) KEGG enrichment analysis was performed for overlapping genes.

The GSE128435 dataset was sourced from the GEO repository. In this dataset, there were 1944 differentially filtered mRNAs; the list encompassed 1026 genes that were overexpressed and 918 genes that were underexpressed. Simultaneously, an exploration of the miR-542-3p target mRNAs was conducted via the starBase repository. The mRNA data from GSE128435, as well as that retrieved from the starBase database, were subsequently depicted using a Venn diagram for visualization (Figure 5A). Subsequently, we performed GO and KEGG analyses on the common mRNAs. The GO functions revealed that the biological processes are primarily associated with the regulation of cellular morphogenesis, positive regulation of neuron differentiation, and response to oxidative stress (Figure 5B). The key cellular components mainly include the RNA polymerase II transcription regulator complex, synaptic vesicle, and transport vesicle (Figure 5C). The primary molecular functions encompassed are protein C-terminus binding, hormone receptor binding, and Ras GTPase binding (Figure 5D). Upon KEGG analysis, it was discovered that the key pathways implicated comprised the neurotrophin signaling pathway, cell cycle, cellular senescence, and human papillomavirus infection (Figure 5E).

### miR-542-3p Regulates the Expression of MYH9

**Figure 6.**
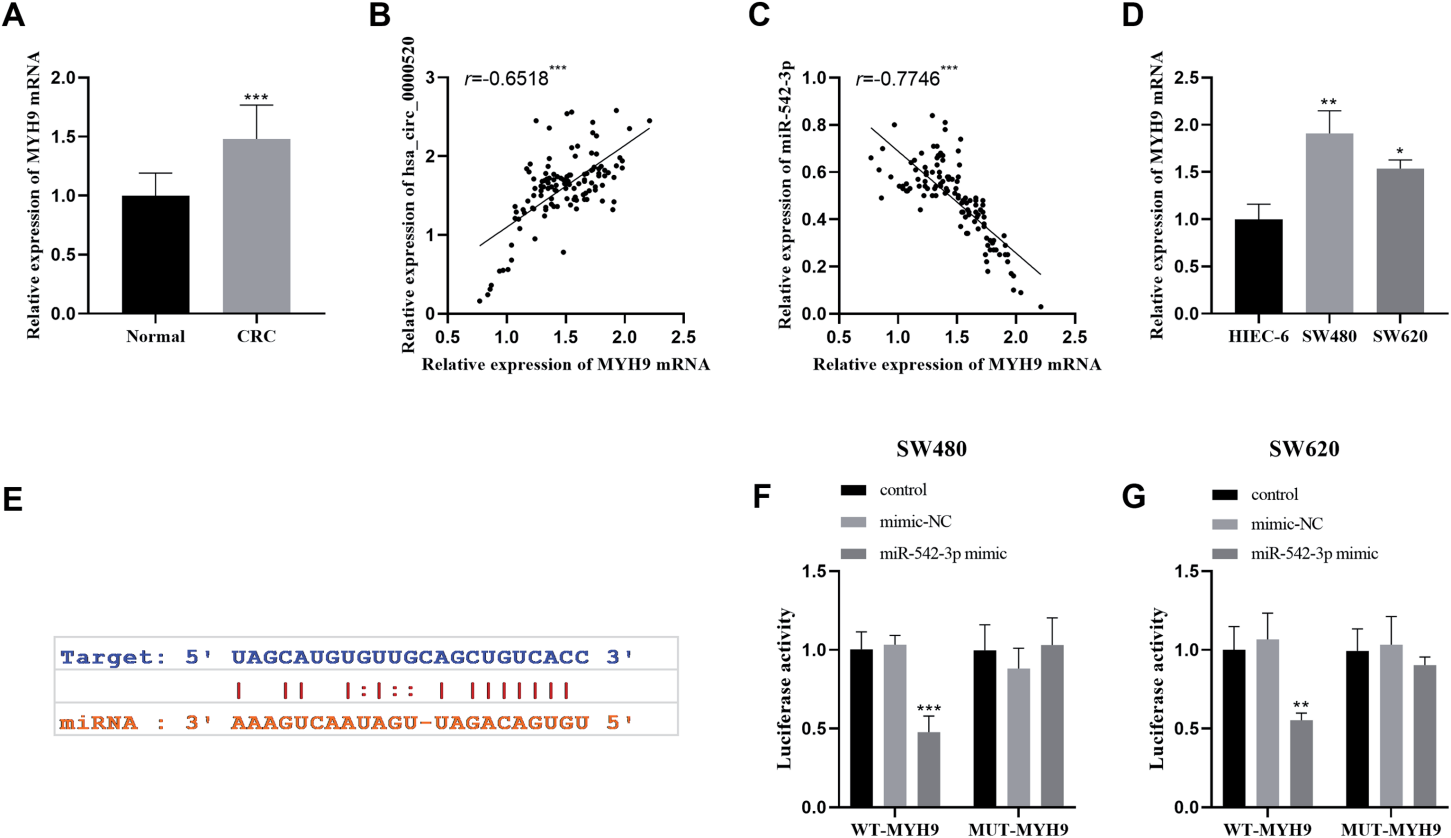
MYH9 is a target gene of miR-542-3p. (A) The level of MYH9 in CRC tissues was detected by qRT-PCR. (B) The expression of MYH9 and hsa_circ_0000520 in tumor tissues was positively correlated. (C) The expression of MYH9 and miR-542-3p in tumor tissues was negatively correlated. (D) The expression of MYH9 in CRC cell lines was detected by qRT-PCR. (E) The sequence of the binding site between MYH9 and miR-542-3p was forecasted utilizing starBase. (F-G) The specific interaction between MYH9 and miR-542-3p was investigated using a dual luciferase reporter gene assay. ^**^P < .01. ^**^P < .01, ^***^P < .001.

MYH9 expression was upregulated in CRC tissues (Figure 6A) and correlated positively with circ_0000520 (Figure 6B) and negatively with miR-542-3p (Figure 6C). Furthermore, MYH9 expression was upregulated in CRC cell lines (Figure 6D). A substantial decrease in luciferase activity was detected in cells treated with miR-542-3p and WT-MYH9 (Figure 6 E-G).

## Discussion

Various investigations have revealed that the levels of circRNAs exhibit altered expression within CRC. For instance, the analysis by Tian and colleagues^18^ led to the identification of 13 198 differentially expressed circRNAs (DECs), which consisted of 6697 that were upregulated and 6501 that were downregulated.

Circular RNAs play crucial roles in the processes of tumor formation, recurrence, and the development of multidrug resistance through a variety of mechanisms.^19^ The upregulation of circRNA_0001666 is linked to a better clinical prognosis for patients suffering from CRC.^22^ The tumor-suppressive function of circRHOBTB3 is achieved through its secretion via exosomes, which contributes to the maintenance of CRC cell viability.^23^ This study indicated that the level of hsa_circ_0000520 is increased in CRC tissues and cells, which is consistent with previous research.^16,17^ In addition, increased expression of hsa_circ_0000520 in tumor specimens was significantly correlated with the clinical characteristics of the patients. This evidence indicated that hsa_circ_0000520 exhibited a strong correlation with the pathological condition of patients with CRC. Additionally, the high level of hsa_circ_0000520 was associated with reduced overall survival and DFS rates, indicating that this circRNA was a factor for poor outcomes in patients. The reduction of hsa_circ_0000520 expression suppressed the proliferation, migration, and invasive capabilities of cells, thus participating in the disease progression of CRC.

Increasingly, research indicates that circRNAs function as miRNA sponges.^24^ In our investigation, we adopted a combined approach of bioinformatics predictions to identify miR-542-3p as a candidate for subsequent research. miR-542-3p in serum functions as a biomarker for osteosarcoma.^27^ miR-542-3p exerts its influence on cellular proliferation control mechanisms in esophageal cancer.^28^ The study revealed that miR-542-3p restrained the proliferation and migration of CRC cells.^29^ Our research identified a decrease in miR-542-3p levels within CRC cell lines, following the same trend as in this study.^29^ Additionally, hsa_circ_0000520 acted as a miR-542-3p binding sponge in CRC cells. The findings implied that hsa_circ_0000520 exerted its influence on cellular processes by modulating miR-542-3p and was consequently implicated in the progression of CRC.

Bioinformatics methods were used to predict the pathways by which mRNA might be involved in CRC. The pivotal signaling pathways identified included the neurotrophin signaling pathway, cell cycle, cellular senescence, and human papillomavirus infection. Multiple studies have reported that genes influence the progression of CRC by influencing the cell cycle, such as Diversin, which augments the proliferative potential of CRC cells through the modulation of cell cycle proteins.^30^ RYBP facilitates a better prognosis in CRC by controlling the cell cycle.^31^ Additional research has revealed that the phenomenon of cellular senescence may contribute to the onset and progression of CRC, suggesting it as a potential therapeutic focus for combatting the illness.^32^ Cellular senescence of CRC cells induced by type 2 diabetes can promote tumor progression.^33^ Based on a comprehensive analysis, an elevated risk of CRC is observed in individuals with a positive result for human papillomavirus infection.^34^

The contractile protein known as MYH9, which is classified as non-muscle myosin IIA, functions as an actin-binding protein, has a significant impact on promoting tumor development, and is strongly associated with a negative outcome for patients suffering from CRC.^36^ In the present investigation, to explore the effector molecules of miR-542-3p, an exploration of the starBase database revealed that MYH9 is a gene targeted by miR-542-3p. The data showed that MYH9 binds specifically to miR-542-3p. This provides stronger evidence that circ_0000520 is involved in the molecular mechanisms of CRC.

Overall, this study provided an important basis for the clinical application of hsa_circ_0000520 in CRC and suggested that hsa_circ_0000520 may be used as a prognostic marker for CRC.

### Limitations

However, the analysis and conclusions drawn from our study do have some inherent limitations. The results of in vivo and in vitro experiments may differ due to the tumor microenvironment and other reasons. Therefore, it is necessary to perform in vivo experiments to provide a more complete basis for the conclusions of this study. Our investigation has shown that hsa_circ_0000520 facilitated the progression of tumors by modulating the expression of miR-542-3p; however, there may be other miRNAs interacting with hsa_circ_0000520 in CRC. Moreover, hsa_circ_0000520 might exert its influence on CRC via additional mechanisms. Consequently, additional research is essential to fully grasp the implications of hsa_circ_0000520 in the emergence of CRC.

## Conclusion

Levels of hsa_circ_0000520 were markedly elevated in CRC cells and patient tumor tissues, and this upregulation was linked to an unfavorable patient survival rate. hsa_circ_0000520 regulates CRC cell function through the miR-542-3p/MYH9 axis, thereby enhancing the aggressiveness of the disease. circRNA_hsa_circ_0000520 emerges as a prospective strategy for the management of CRC cases.

## Availability of Data and Materials

The data that support the findings of this study are available on request from the corresponding author.

## Figures and Tables

**Table 1.** Correlation Between the Tumor Tissue Expression of hsa_circ_0000520 and Clinical Indicators in Colorectal Cancer Patients

Parameters		Patients (n = 119)	Low hsa_circ_0000520 expression (n = 52)	High hsa_circ_0000520 expression (n = 67)	*P*
Age (years)	<60	62	25	37	.439
	≥60	57	27	30	
Gender	Male	63	26	37	.571
	Female	56	26	30	
Tumor size (cm)	<4	65	32	33	.182
	≥4	54	20	34	
TNM stage	Ⅰ-Ⅱ	65	36	29	.005^**^
	Ⅲ-Ⅳ	54	16	38	
Lymph node metastasis	No	79	42	37	.003^**^
	Yes	40	10	30	
Tumor differentiation	Poor	73	26	47	.025^*^
	Moderate/high	46	26	20	
Tumor location	Colon	65	26	39	.372
	Rectum	54	26	28	

^*^P < .05; ^**^P < .01.
